# A Hypothesis and Review of the Relationship between Selection for Improved Production Efficiency, Coping Behavior, and Domestication

**DOI:** 10.3389/fgene.2017.00134

**Published:** 2017-09-28

**Authors:** Wendy M. Rauw, Anna K. Johnson, Luis Gomez-Raya, Jack C. M. Dekkers

**Affiliations:** ^1^Departamento de Mejora Genética Animal, Instituto Nacional de Investigación y Tecnología Agraria y Alimentaria, Madrid, Spain; ^2^Department of Animal Science, Iowa State University, Ames, IA, United States

**Keywords:** coping styles, domestication, feed efficiency, genetic selection, life-history theory, resource allocation

## Abstract

Coping styles in response to stressors have been described both in humans and in other animal species. Because coping styles are directly related to individual fitness they are part of the life history strategy. Behavioral styles trade off with other life-history traits through the acquisition and allocation of resources. Domestication and subsequent artificial selection for production traits specifically focused on selection of individuals with energy sparing mechanisms for non-production traits. Domestication resulted in animals with low levels of aggression and activity, and a low hypothalamic–pituitary–adrenal (HPA) axis reactivity. In the present work, we propose that, *vice versa*, selection for improved production efficiency may to some extent continue to favor docile domesticated phenotypes. It is hypothesized that both domestication and selection for improved production efficiency may result in the selection of reactive style animals. Both domesticated and reactive style animals are characterized by low levels of aggression and activity, and increased serotonin neurotransmitter levels. However, whereas domestication quite consistently results in a decrease in the functional state of the HPA axis, the reactive coping style is often found to be dominated by a *high* HPA response. This may suggest that fearfulness and coping behavior are two independent underlying dimensions to the coping response. Although it is generally proposed that animal welfare improves with selection for calmer animals that are less fearful and reactive to novelty, animals bred to be less sensitive with fewer desires may be undesirable from an ethical point of view.

## Introduction

Coping refers to the individual’s behavioral and physiological efforts to manage (reduce, minimize, master, or tolerate) the internal and external demands of a situation that is appraised as stressful, and taxing or exceeding the individual’s resources (Folkman et al., 1986; Koolhaas et al., 1999). The ability to adequately cope with stress is directly related to individual fitness in natural populations and to animal welfare in production animals. Selection for production traits may positively or negatively influence the individuals’ coping capacity and therefore its welfare. It is the aim of the present work to review coping behavior in humans and animals, describe its relationship with other fitness traits, and hypothesize on the consequences of selection for improved production efficiency on coping behavior.

## Coping in Animals

### Coping in Humans

As extensively reviewed by Endler and Parker (1990), Lazarus (1993a), and Suls and David (1996), in humans, interest in coping concepts became more prominent during the 1960s and 70s as interest in the study of stress increased. In the early years, coping was conceptualized as a psychological style or inner (primarily unconscious) psychodynamic response in defense of personal (“ego”) integrity and threat management. For example, Sigmund Freud described 10 defense mechanisms: regression, repression, reaction formation, isolation, undoing, projection, introjection, turning against the self, reversal, and sublimation (in Freud, 1992). Some defense styles were considered healthier or less regressed than others (Lazarus, 1993a). Other researchers formulated the ego defenses as trait-like coping styles. For example, Byrne proposed a continuum between repression, i.e., behavior mechanisms of a predominantly avoiding (denying, repressing) type, and sensitization, i.e., behavior mechanisms of predominantly approaching (intellectualizing, obsessional) behaviors (Byrne, 1961; Suls and David, 1996). Some 13 additional formulations on the approach-avoidance scale are reviewed by Roth and Cohen (1986).

In contrast, the coping *process* approach developed by Lazarus and colleagues in the 1970s and 80s regarded coping strategies to be a function of the situational context in which it occurs, as influenced by external environmental forces (Lazarus, 1993a). According to this approach, coping processes change over time and are not based on preconceived notions of inherent health and adaptiveness or pathology and maladaptiveness. This approach emphasizes two major functions of coping: “problem” focused coping, i.e., to actively attempt to change the environment by removing the source of stress or remove oneself from the source of stress, versus “emotion focused” coping, i.e., to reduce the emotional impact or the negative appraisal of stress (Folkman and Lazarus, 1980). In particular, emotion focused coping may predominate in situations that are perceived to be “refractory to change,” whereas problem focused coping may predominate in situations that are perceived as “controllable by action” (Lazarus, 1993a). However, it became empirically clear that the situational context does not account for all of variation in coping behaviors (Suls and David, 1996). In the 1980s and 1990s, research revealed that coping strategies may be influenced by personality variables, such as the tendency to be optimistic vs. pessimistic (Scheier et al., 1986). In fact, it was reported that some 30% of the variation in single-act behaviors can be explained by personality traits and a considerably larger portion may be explained when behavioral cross-situational aggregates rather than situation-specific single-act behaviors are used (Kenrick and Funder, 1988). Indeed, Kato and Pedersen (2005) observed that genetic influences on coping scales were partly attributable to genetic factors that are associated with personality traits.

Therefore, to some extent, coping strategies were shown to be consistent, showing a stable pattern over time and across stressful encounters and could be viewed as a stable coping disposition or style. A well-described coping style is called the Type A pattern, which Friedman and Rosenman (1974) defined as “an action–emotion complex that can be observed in any person who is aggressively involved in a chronic, incessant struggle to achieve more and more in less and less time, and if required to do so, against the opposing efforts of other things or other persons” (in Matthews, 1982). As reviewed by Matthews (1982), this behavioral style can be regarded as a continuum of behaviors ranging from the extreme Type A to the opposite extreme Type B. Type A personalities are characterized as having increased levels of competitiveness, aggressiveness, achievement striving, ambition, impatience, and hostility (Heilbrun and Renert, 1986). In response to stress, Type A personalities show an active response and a greater tendency to rely on achievement related, solution-oriented, problem-focused coping, directed at bolstering the perception of controllability (Evans and Fearn, 1985; Hart, 1988). In the 1980s, personality dimensions were extended to broader sets of personality dimensions, such as “the Big Five”: Neuroticism (N) vs. Emotional Stability; Extraversion (E) or Surgency; Openness to Experience (O) or Intellect; Agreeableness (A) vs. Antagonism; and Conscientiousness (C) or Will to Achieve (Costa and McCrea, 1992), which are also associated with stress-related coping processes (Penley and Tomaka, 2002).

Speisman et al. (1964) observed that “control of the environment” was an important modulating factor that determines stress response characteristics. Indeed, the interaction between behavioral type (A or B) with “locus of control” significantly influenced manager work satisfaction and health outcomes (Kirkcaldy et al., 2002). Managers with external locus (believing that events in their lives are a function of luck, chance, fate, God(s), or powerful others) showed significant lower job satisfaction levels and more negative health consequences than managers with internal locus (believing that events are a function of their own behavior and/or ability, personality, or effort), especially when this characteristic was combined with a Type A personality. Speisman et al. (1964) identified that controllability was related to separate catecholamine (adrenaline and noradrenaline) and cortisol factors. The catecholamine factor is predominantly related to coping “effort,” whereas the cortisol factor is predominantly related to coping “distress,” i.e., cortisol tends to increase in novel and unfamiliar situations that evoke feelings of uncertainty and anxiety. Comparing response to a low-control and a high-control task, they observed that during the high-control task adrenaline, but not cortisol, increased (effort without distress), whereas during the low-control task both adrenaline and cortisol increased (effort with distress). In addition, Speisman et al. (1964) linked personality, perception of control, and situational context, in that together they form the threat *meaning* or “harmful significance” of an event. Indeed, “Stressors, like beauty, lie in the eye of the beholder,” i.e., it is only when a situation is *perceived* as a potential threat that a stress response is initiated (Everly and Lating, 2013). For example, an event may be perceived as an unpleasant threat but also as an exhilarating challenge, and resulting from the motivation toward the event, both the appraisal of the event and the subsequent coping response can be described as a function of stress emotional states, including anger, anxiety, guilt, shame, sadness, envy, jealousy, and disgust, but also happiness, pride, relief, love, hope, compassion, and gratitude (Lazarus, 1993b). Frankenhaeuser and Lundberg (1985) confirmed that catecholamine output under different psychosocial conditions was linearly related to the intensity of the emotional experience (and consequently the degree of *subjective stress*), whether pleasant or unpleasant.

### Coping in Non-human Animals

Methods used for coping assessment in animals are fundamentally different from those used in human research. As reviewed by Parker and Endler (1992), coping research in humans is primarily based on various self-report coping scales and measures in response to (the thought of) a stressful event, such as the Ways of Coping Questionnaire (Folkman and Lazarus, 1988). Because animals cannot directly communicate their cognitive emotional appraisal, motivation, or behavioral approach toward a stressful event these questionnaires have no use in animal research. Instead, the behavioral and/or physiological response to social and non-social situations is evaluated. However, although coping assessment methods are inherently different in animals and humans, research in coping strategies in humans and animals has, with few (notable) exceptions (Zozulya et al., 2008; Cavigelli et al., 2013), surprisingly little overlap in its discussion and implications of the results. Whereas human research is based on (unconscious or conscious) psychological coping mechanisms that are thought to be influenced by personality traits, since assessment methods in animals are unable to evaluate the latter, coping in animal research is described by behavioral and physiological responses that are themselves equated to a personality concept. Based on earlier work studying individual differences between animals during defensive behavior, it was hypothesized that two fundamentally distinct coping styles exist: animals that respond to social interaction with an active fight–flight response that is characterized by a behavioral and neuroendocrine response highly suited to either attack or flight, versus animals that respond with a passive conservation-withdrawal response that is characterized by a neuroendocrine response resulting in behavioral inhibition (Benus et al., 1992). Blanchard et al. (2011) discussed fight–flight vs. conservation-withdrawal strategies in a social confrontation from a process approach as a function of the situational context in which it occurs, as influenced by external environmental forces: situational risk assessment (i.e., appraisal through auditory, visual, and olfactory detection) shapes the defensive response choice on a likelihood of success prediction compared with taking a different approach. For example, an animal may flight if an escape route is available, hide if there is a place of concealment, freeze if there is neither an escape route nor a hiding place, show defensive threats as the threat stimulus approaches, and defensive attack when contact with the threat stimulus is imminent. Indeed, the distance between the subject and the threat was found to be a rather precise determinant of freezing (longer distances) vs. fight behavior (shorter distances) (Blanchard et al., 2011). In contrast, similar to humans, a coping *style* is defined as a coping strategy that is consistent when measured repeatedly in the same situation, as well as in response to different situations, i.e., (at least to some degree) independent of the situational context (Koolhaas et al., 1999). In a social confrontation, fight–flight-type animals are more aggressive, attack, or actively try to escape when defeated, and are characterized by a predominantly sympathetic adrenal-medullary response pattern, whereas conservation-withdrawal-type animals are less aggressive, more immobile, hardly respond to attacks, and respond with a parasympathetically dominated response and greater adrenocortical activity. Indeed, Koolhaas et al. (1999) showed that mouse lines divergently selected for aggressive social behavior (attack latency) were representative of two distinct coping styles: proactive, aggressive (fight–flight type) animals and reactive, non-aggressive (conservation-withdrawal type) animals (Benus et al., 1991). In addition to differences in social behavior, the lines also differed with respect to other behavioral components. As opposed to reactive animals, proactive animals are fast exploring, impulsive, actively manipulate events, score high in frustration tests, and are risk takers and novelty seekers (Benus, 1988; Coppens et al., 2010). Overall, aggressive mice were better shock avoiders (Benus et al., 1989) and had greater mobility in a novel object test (Caramaschi et al., 2009). They tended to react in a rather routine way to changes in the environment (i.e., intrinsic behavioral control), whereas non-aggressive individuals seemed to be more attentive to their environment (i.e., extrinsic behavioral control) (Benus et al., 1987). Further research showed that also the social response of aggressive mice toward a conspecific was more routine-like and inflexible than that of non-aggressive mice (Benus et al., 1990).

Coping styles in rodent studies are extensively reviewed by Cavigelli et al. (2013). After the rodent studies, coping styles were reported in other species as well, in particular in animals managed by humans, such as pigs (Bolhuis et al., 2005), chickens (Jensen et al., 2005), cattle (Hopster, 1998), fish (Øverli et al., 2004), horses (Budzyńska, 2014), dogs (Horváth et al., 2007), and cats (Natoli et al., 2005), but also in natural species such as birds (Carere et al., 2001), marmots (Costantini et al., 2012), wild rabbits (Rödel and Monclús, 2011), chipmunks (Montiglio et al., 2012), salamanders (Crane et al., 2012), crickets (Kortet and Hedrick, 2007), and spiders (Johnson and Sih, 2005). It is now clear that variation in behavioral traits has a clear genetic basis and that behavioral traits do not inherit independently of each other (Van Oers et al., 2005). Because individual behavioral traits within a style are genetically correlated, (divergently) selecting for extremes of one behavioral style trait, such as aggression in the studies of Koolhaas et al. (1999), will result in a correlated response in other style traits. For example, Sluyter et al. (1995) showed that mice selected for short attack latency showed more nest-building behavior than those selected for high attach latency, whereas mice in a reciprocal selection experiment selected for high nest-building activity were more aggressive than those selected for low nest-building activity. Annen and Fujita (1985) showed that rats selected for “low emotional reactivity” (i.e., high ambulation and low defecation scores in a brightly lit runway) were also more aggressive than those selected for “high emotional reactivity or timidity,” and mink selected for confident reaction to humans showed more exploratory behavior across several social and non-social situations than mink selected for fearful reaction toward humans (Malmkvist and Hansen, 2002). Mice selected for high wheel-running behavior (voluntary physical activity) were more explorative in the open-field test, and showed more risk-taking behavior in approaching a novel object relative to a control line (Jónás et al., 2010). Great tits selected for slow exploratory performance took longer to attack than those selected for fast exploratory performance (Carere et al., 2005).

### Coping in Livestock Animals

Livestock animals require to cope with the demands of a semi-natural production environment that includes few features that the individual can manipulate or change (Wechsler, 1995). Several studies have indicated that the behavioral organization of livestock has not been changed to a great extent by domestication; therefore, intensive housing conditions are likely to trigger behavioral and physiological coping efforts in response to aspects including limited space allowance, absence of key stimuli, and semi-natural feeding regimes and social group compositions (Duncan, 1998; Bracke and Hopster, 2006). Because unsuccessful coping is closely related to the development of abnormal (stereotypic) behavior, coping ability in livestock animals directly relates to the individual’s ability to adapt to specific management conditions and therefore animal wellbeing (Wechsler, 1995). In this context, coping research in livestock species is mostly directed to evaluation of the response to novel environmental cues, and (anti)social behavior.

Hessing et al. (1993) classified piglets in response to a restraint backtest as resistant, doubtful, or non-resistant. A high consistency in their behavioral response was observed in successive restraint tests, in addition, individuals that resisted in the backtest were also classified as “aggressive” individuals immediately after relocation and mixing, whereas individuals that did not resist were classified as “non-aggressive” individuals. The authors indicated that the results may be particularly important in realizing the optimum group composition based on individual characteristics. Indeed, Bolhuis et al. (2005) showed that resistant pigs initiated more fights, started fighting earlier, and spent more time fighting after regrouping. In addition, resistant pigs appeared to have a higher propensity to develop inflexible behavioral routines (Bolhuis et al., 2004). D’Eath and Burn (2002) and Cassady (2007), however, did not find any relationship between resistance in the backtest and aggressive behavior in a resident–intruder test in piglets. Neither did Janczak et al. (2003), who observed repeatability in response to specific or closely related stimuli, but did not find any correlation between resistance to the backtest, and a resident-intruder, human in the home cage, or novel object test.

The conclusion was shared by Herskin et al. (2004) in dairy cows, who observed stimulus specificity in the reactions of dairy cows toward different novel object tests. Also Gibbons et al. (2009) observed consistency over time in flight response scores and consistency between different human approach situations, but no consistency was found between human approach situations and novel object test scores. However, Müller and Von Keyserlingk (2006) observed that beef cattle having higher temperament as measured by repeated measurements of flight speed from a squeeze chute were more agitated during a social separation test as measured by locomotion and behavioral states. In rangeland-raised beef cattle, Wesley et al. (2012) observed consistent behavioral styles in foraging behaviors across contexts (confinement vs. rangeland pasture), and also MacKay et al. (2013) observed a correlation between observations in a short-term temperament test and aggression at feeders.

Korte et al. (1998) observed that chicks from a low feather pecking line of laying hens had a higher parasympathetic activity in response to manual restraint than chicks from a high feather pecking line, and concluded that individuals of both lines were passive and active, respectively, in terms of coping style. This was supported by Jensen et al. (2005), who observed that feather pecking behavior in chickens was positively correlated with activity in an open-field test, novel food/novel object test, and in a restraint test, which suggests that feather pecking might be genetically linked to a proactive coping style. However, Jones et al. (1995) observed that individuals from a chicken line selected for high feather pecking showed more freezing, and less vocalization and activity in an open-field, which is more indicative of a reactive coping style.

It has been proposed that lack of repeatability of intra-test results may result when tests are more situation-specific and therefore less indicative of true differences in individual stress-coping behavior measurable throughout an individual’s life (Cassady, 2007). Lack of correlations between both intra- and inter-test results have been proposed to result from differences in stability of underlying emotions, or the perception of or the biological motivation toward certain tests (Ruis et al., 2000). In addition, Ruis et al. (2000) indicated that the (dynamics of) group compositions may affect the individual coping response of animals and as a result the correlations between results and the interpretation of data.

## Coping Style as a Life-History Trait

### Adaptation and Survival

A renewed interest in coping styles has emerged in the fields of ecology, evolutionary, and natural biology, with a focus on the structure of coping styles, its proximate and ultimate causes, its relation to life-history strategies, and consequently its ecological and evolutionary significance (Wolf and Weissing, 2012; MacKay and Haskell, 2015). Thus far, research into coping behavior in the context of natural evolution had followed the coping process approach, under the understanding that behavioral strategies were potentially infinitely plastic and, therefore, natural selection favored different optimal strategies in different situational contexts (Sih et al., 2004; Bell, 2007). In the ecological literature, coping style, or within-individual consistency in behavior, is also referred to as “behavioral type”; proactive, aggressive copers are referred to as “bold types” and reactive, non-aggressive copers as “shy types.” In addition, a new dimension has been added, the behavioral *syndrome*, which refers to *between*-individual consistency in behavior across multiple situations, or “the correlation of rank-order differences between individuals over time and/or across situations” (Bell, 2007). Behavioral syndromes are thus a property not only of the individual, but also of the population, since they describe the distribution of behavioral types (or coping strategies) within a population (MacKay and Haskell, 2015). As reviewed by DeWitt et al. (1998), adaptive phenotypic plasticity infers costs such as those related to the production and maintenance of sensory and regulatory mechanisms of plasticity, and information acquisition. However, the cost and benefits of limited plasticity, as implied by the existence of behavioral syndromes, may trade-off with the ability to adapt to a variety of environments. Therefore, the existence of coping styles implies that individuals may be adapted to a certain environment but display behaviors that are suboptimal in a different one. For example, proactive copers rely mainly on internally organized predictions, which may be fast but also inaccurate in new situations, whereas passive copers are more guided by external stimuli than active copers and hence their behavior is more flexible and adaptable to variable and unpredictable environmental conditions (Benus, 1988; Coppens et al., 2010). Similarly, too high levels of aggression may be unsuitable in contexts where caution and care may be more appropriate, but unaggressive individuals may do poorly in competitive situations (Sih et al., 2004). However, although the flexibility or variety of behavioral strategies employed by an individual in different situations is, to some extent, restricted (behavioral types), the magnitude of the response in different situations may be flexible (behavioral syndrome), while consistently differing from that of other individuals in the population (Biro and Stamps, 2008).

Coping styles are closely related to individual fitness and are part of life-history strategies, since they are related to risk-taking behavior and form general adaptive response patterns in reaction to everyday challenges and stress (Coppens et al., 2010). In addition, activity, exploration, boldness, and aggression are energetically costly. Life history is commonly defined as a set of evolved behavioral and physiological strategies that more or less influence longevity and reproduction and may include fitness traits such as reproductive success, survival, viability, fecundity, mating success, and age at maturity (Schluter et al., 1991). A fundamental assumption of life-history theory is that resources are limited and need to be distributed among growth, reproduction, and maintenance, or stored for future use. Since resources used for one purpose are no longer available for other purposes, trade-offs are inevitable. Therefore, coping styles can be expected to trade off against other life-history traits (Wolf et al., 2007; Wolf and Weissing, 2012). Natural selection results in the optimal allocation of resources across important life-history functions (Brommer, 2000; Roff, 2007). Since nature has not favored any coping strategy in particular, both strategies may have benefits and should be considered as alternative strategies to cope with environmental demands (Coppens et al., 2010). More recently, behavioral syndrome theory has been integrated in the life-history pace-of-life syndrome hypothesis, thus mapping behavioral styles on the “slow” (low metabolic rate, slow development, late maturation, long life span, high investment in few offspring) to “fast” (high metabolic rate, fast development, fast maturation, low survival, low investment in many offspring) pace-of-life scale, and connecting them with a series of metabolic, hormonal, and immunity traits that underlie this syndrome (Réale et al., 2010). Indeed, several empirical studies provide evidence for links between behavioral types and food intake, growth, and reproductive traits (Biro and Stamps, 2008). Bold individuals with high levels of aggression may have better access to resources necessary for fast development and early reproduction, and have more reproductive success, but this may also be associated with a higher risk of mortality. Indeed, Smith and Blumstein (2008) showed in a meta-analysis that bolder individuals have greater reproductive success but this incurs a survival cost. Thus, shy individuals that have reduced short-term reproductive success but live longer may have the same overall fitness as bold individuals.

### Health

The Type A behavior pattern in humans arose from the observation by Friedman and Rosenman that patients with cardiac disorders seemed to have different behavioral characteristics than noncardiac patients. Meanwhile, Type A as an independent risk factor for coronary heart disease (CHD) has been firmly established (Matthews, 1982). However, the nature of the link with Type A behavior was not immediately recognized, partly because the definition of Type A behavior is broad and complex and has been described (and sometimes misclassified) in a wide variety of ways (Friedman and Booth-Kewley, 1987). As proposed by Lazarus (1993b), it appears that it is the stress emotional state that determines whether individuals classified as Type A are prone to CHD while those classified as Type B are not (Friedman and Booth-Kewley, 1987). For example, it appears that emotional reactions of impatience, hostility or repressed hostility, anger, aggression, and tenseness are characteristic of the CHD-prone person. Therefore, hostile, competitive, aggressive striven Type A individuals may be prone to CHD but not active, hard-working, confident, charismatic, socially skilled, dominant, vigorous, ambitious individuals. Likewise, relaxed, easygoing, reserved Type B individuals may not be prone to CHD, but individuals that experience strong emotions but have some difficulty in expressing them openly may be prone to CHD. In addition, depression and anxiety appear to be underlying risk factors for CHD that are not always clearly expressed in the expected behavioral type (Diamond, 1982; Friedman and Booth-Kewley, 1987). Folkman et al. (1986) showed that individuals who display more confrontive coping (e.g., “stood my ground and fought for what I wanted”), distancing (e.g., “went on as if nothing had happened”), self-controlling (e.g., “I tried to keep my feelings to myself”), accepting responsibility (e.g., “criticized or lectured myself”), and escape avoidance behaviors (e.g., “wished that the situation would go away or somehow be over with”) had a lower somatic health status, whereas individuals that felt more “mastery” (i.e., that regard one’s life chances as being under one’s control in contrast to being fatalistically determined) were healthier. However, Folkman (1984) indicates that in humans, being in control may not always be stress reducing and result in a positive situational appraisal.

Also in other animals, proactive and reactive individuals are found to differ in the physiological and neuroendocrinological response to stress, which may have implications for their health status. Several studies, as reviewed by Koolhaas (2008), demonstrate that the nature of the neuroendocrine stress response may modulate the immune response and individual vulnerability to disease. The response of proactive animals is often found to be dominated by an enhanced sympathetic and (nor-)adrenergic response, resulting in high plasma levels of adrenalin and noradrenalin and a high heart rate and blood pressure, whereas the response of reactive animals is dominated by enhanced parasympathetic activation and a high hypothalamic–pituitary–adrenal (HPA) response, resulting in a bradycardia response in reaction to a sudden unpredicted stressor and increased plasma levels of corticosteroids. However, when defeated in a social confrontation, proactive animals appear to respond with a corticosterone response that is higher than that of defeated reactive mice (Carere et al., 2001; Koolhaas, 2008). Morrow-Tesch et al. (1994) showed that both socially dominant and submissive pigs were immune compromised (elevated numbers of neutrophils, decreased antibody production) compared with socially intermediate pigs and concluded that although dominance may afford the animal greater priority to resources (like food and mates), it may have some immunosuppressing effects as well.

The reactivity of the stress response is shaped both by genetics as well as by gene × environment interactions resulting in epigenetic modifications (Rauw and Gomez Raya, 2017). Kadarmideen and Janss (2007) estimated that cortisol in a pig selection experiment is highly genetically determined with heritabilities of 0.40–0.70. They observed that cortisol levels are determined by a mixture of genes with large and small effects, but also detected a major gene with an additive effect of 86 ng/ml. Sautron et al. (2015) identified 65 genes in pigs as biological markers of HPA axis activation at the gene expression level. In chickens, Fallahsharoudi et al. (2017) detected one genome-wide significant QTL on chromosome 5 and two suggestive QTL which together explained 20% of the variance in corticosterone response.

### Energetic Trade-Offs

Although energetics is a fundamental assumption of life-history theory, surprisingly little is published to date on theory or observation linking behavioral styles and syndromes to energy budgets and resource allocation. However, because exploration, boldness, and aggression are energetically costly, from a resource allocation point of view it can be expected that different coping strategies may constitute different metabolic costs and present different trade-offs with growth, reproduction, and immune function. For example, depending on body weight, a minimum cost can be defined for such activities as walking, running, flying, and swimming (Tucker, 1970; Videlex and Nolet, 1990). Fighting depletes energy reserves and (anticipated) metabolic consequences may determine strategic decision making during social interaction (Neat et al., 1998). In fish, Neat et al. (1998) showed that escalated fighting is costly for both winners and losers, but especially for losers. Indeed, aggressive disposition may be beneficial if it allows for greater food intake and faster growth. Similarly, for bold individuals, the benefits in terms of dispersal potential and gaining access to new resources may be offset by increased energetic costs from higher metabolic rates when at rest (Myles-Gonzales et al., 2015). Indeed, when food cannot be monopolized, the greater costs of behaviors associated with an increased standard metabolic rate may significantly reduce growth (Vøllestad and Quinn, 2003; Georgiev et al., 2013). Careau et al. (2010) found that aggressive dog breeds have higher energy needs than unaggressive ones and, in addition, docile dogs live longer than bold ones. Biro and Stamps (2010) reviewed literature that reported significant positive relationships of several types of behavior (aggressive behavior, success in competitive interactions, activity rates, boldness, scrounging, courtship) with resting metabolic rate and therefore maintenance cost in a diverse array of taxa.

Because behaviors underlying fight–flight behavior are fueled by a greater metabolic rate, it may appear that fight–flight coping styles are costlier than conservation–withdrawal styles. However, the multi-axial physiological stress response may also demand a significant amount of resources. Indeed, the concept of allostasis, i.e., energy demanded by physiological mediators or “homeostats” of the physiological stress response is deeply integrated into the concept of life history theory and trade-offs through resource allocation (Goldstein, 2003; Rauw and Gomez Raya, 2017). One of the best known physiological mediators is the HPA axis, which functions as a primary mediator of energy balance homeostasis, as a master organizer of life-history transition, and as an integral responder to stressors (Crespi et al., 2013). Glucocorticoid-induced physiological changes include gluconeogenesis for providing energy to fuel the greater metabolic demands, and the stimulation of feeding behavior to replenish depleted energy stores following a stress response (Matteri et al., 2000). Resources used by this and other physiological mediators determine the total energy demanded or “allostatic load,” which is a function of the response to metabolic demand of daily and seasonal routines and of unpredictable stressors (Romero et al., 2009). When stimulation of the mediators is prolonged or severe, such that costs are larger than those available in the reserve, resources must be reallocated away from other biological functions, which then become impaired (Moberg, 2000). For example, increased costs associated with the stimulation of the HPA axis result in reduced growth rates in all livestock species (Rauw and Gomez Raya, 2015). In addition, glucocorticoids are largely immunosuppressive, which could be an adaptive mechanism to reallocate resources from activation of the immune defense system (Råberg et al., 1998). Therefore, the reduced cost of the coping response of reactive individuals that result from reduced levels of activity and aggression may (partly) be offset by increased energetic costs associated with a higher HPA response.

## Coping Styles and Domestication

In their famous fox experiment, Belyaev et al. (1985) showed that domestication meant that individuals were selected for tameness, or tolerance of and reduced aggression toward humans. In addition to unconscious or deliberate selection for the domesticated behavioral non-aggressive, tame phenotype, dogs are proposed to have passed a phase of self-domestication, where less aggressive domesticated phenotypes gained increased access to resources in human settlements. The self-domestication hypothesis has also been proposed for the origin of numerous differences between bonobos and chimpanzees (Hare et al., 2012), and even for our hominid ancestors, resulting in new forms of social interaction and communication (Hare and Tomasello, 2005). Indeed, Hare et al. (2012) propose that self-domestication through natural selection for reduced aggression may have been a widespread process in mammalian evolution. Because of the concomitant systematical down-regulation of aggressive behavior in (self-)domesticated mammals, it can be hypothesized that domestication resulted in the selection of non-aggressive individuals with a more reactive coping style. Indeed, Red Jungle Fowl are found to be more explorative and active compared to domestic chicken breeds (Ericsson and Jensen, 2016). In addition, wild cavies showed more explorative behavior in an open-field, and were more risk-taking when having to jump off an elevated platform than domestic guinea pigs (Zipser et al., 2014). According to Price (1999), the single most important effect of domestication on behavior is possibly reduced sensitivity and increased adaptability to environmental changes, a characteristic that can be observed in virtually all populations of domestic animals. This theory appears to be supported by the observation that both domesticated and reactive individuals show increased serotonin neurotransmitter levels, which plays a role in impulsive aggression at the level of the prefrontal cortex (Coppens et al., 2010). Selection for tameness in silver foxes (Popova et al., 1991b) and low aggressiveness to man in Norway rats (Popova et al., 1991a) has resulted in animals that show a higher serotonin neurotransmitter level in the midbrain and hypothalamus. Selection for low fear of humans in Junglefowl (Agnvall et al., 2015) resulted in males that had higher plasma levels of serotonin. It has therefore been proposed that the brain serotonergic system is involved in the mechanism of domestication, converting wild aggressive/defensive animals into tame ones (Popova et al., 1991b). Also, reactive mice and rats have been shown to have higher serotonin levels than proactive animals (Coppens et al., 2010). A brain circuit in which serotonin neurons moderate coping behavior was recently presented by Puglisi-Allegra and Andolina (2015). Although both reactive and proactive behavioral styles are particularly well described in many domesticated species, the *degree* of behavioral expression may have changed. According to Koolhaas et al. (1999), a bimodal or otherwise non-normal distribution of behavioral styles (mostly latency measures) is found in feral and wild animal populations, whereas several investigations using laboratory strains or livestock animals were unable to find clearly distinct coping styles (including resistance in a backtest). It was proposed that these non-normal distributions may result when intermediate coping styles are less successful in nature and thus have reduced fitness. Alternatively, coping styles of domesticated animals may overlap (or be normally distributed within) the reactive behavioral style of wild ancestors.

Because aggression is an essential part both of behavioral styles and the domestication syndrome, it can be theorized that (self)-domestication of animals through selection of individuals with a “reactive coping style” resulted in concomitant changes both in other behavioral style traits, as well in traits included in the domestication syndrome. The domestication syndrome recognizes phenotypic regularities in the domesticated phenotype, which include neoteny, loss of strict seasonal patterns of reproduction, increased fertility, variations in coat color and texture, docility, alterations in skull shape, and floppy ears (Wilkins et al., 2014). Indeed, compared with chimpanzees, less severe forms of aggression in self-domesticated bonobos also resulted in reduced cranial size, a white tail-tuft, intensified sexual behavior, and more play behavior into adulthood (Hare et al., 2012). However, the domestication syndrome and the reactive coping style do not overlap, indeed appear to be quite opposite, in one important aspect: the HPA axis reactivity to stress, which is responsible for fear, stress, and adaptation. Domestication quite consistently results in a delayed adrenal gland maturation and a decrease in the functional state of the HPA axis, resulting in an extended socialization window, and a relatively immature emotional response to social threat, raising the behavioral thresholds for aggression, fight, and flight (Zeder, 2012; Wilkins et al., 2014). In silver foxes selected for tameness, basal, and stress-induced blood cortisol levels decreased with advancing selection, and had reduced three- and fivefold in generation 45, respectively (Trut et al., 2009). Also in rats selected for tameness, serum corticosterone levels were significantly lower than in rats selected for defensive aggression toward humans (Albert et al., 2008). Künzl and Sachser (1999) showed that the reactivity of the pituitary–adrenocortical system was distinctly reduced in the domesticated guinea pig compared to its wild ancestor the cavy, and Fallahsharoudi et al. (2015) showed that domesticated chickens have a blunted HPA axis reactivity compared to their red junglefowl ancestors. This is in contrast to the coping style of reactive animals, which is often found to be dominated by a *high* HPA response. However, although these differences are quite consistently observed in different species, animals with different coping strategies do not always show this typical stress response (Herborn et al., 2011). For example, Frank et al. (2006) showed that rats that were divergently selected for high or low trait anxiety showed highly divergent reactive and proactive coping behaviors, respectively, however, adrenocorticotropin and corticosterone were secreted to a *higher* extent in the proactive low anxiety line, indicating a dissociation of behavioral and neuroendocrine stress responses. Also, Ferrari et al. (2013) observed that cortisol production in wild marmots was totally independent of behavioral coping styles in reaction to a stressor.

As in humans, this discrepancy may be resolved by distinguishing coping styles from personality variables. In agreement with observations in humans, Koolhaas (2008) proposed that the physiological response is related to individual emotionality rather than the coping style dimension *an sich*. Van Reenen et al. (2005) observed that measures of adrenocortical and behavioral reactivity in an open-field and novel object test were highly consistent over time, however, HPA axis reactivity to ACTH or CRH was unrelated to adrenocortical and behavioral responses to novelty. They suggested that, instead, high cortisol and avoidance responses to novelty reflect underlying fearfulness, and proposed a model that maps the response along two independent underlying dimensions: activity and fearfulness. In addition, the (perception of or hope for) success of the individual in employing a coping strategy to master, reduce, or tolerate the internal and/or external demands that are created by the stressful event may influence the perception and appraisal of the stress event. Indeed, whereas an individual that has a reactive coping style in nature may not necessarily have a reduced perception of the stressor, domestication specifically meant selecting for tameness, i.e., a combination of low aggression *and* low fearfulness. Interdisciplinary research provides evidence that (to some degree) animals experience emotions such as joy, fear, love, despair, and grief (Bekoff, 2000), and have the capacity for episodic memory and future planning (Zentall, 2013). Therefore, whereas human research benefits from the ability of individuals to articulate the dimensions of their coping strategies in terms of their behavioral response but also their emotion, motivation, control, and event appraisal, equating coping response with personality in animal research may delimit our understanding of animal coping strategies.

## Selection for Feed Efficiency in Livestock: Further Domestication?

From an energetic perspective, the process of domestication tended to reallocate resources used for processes that were no longer needed in the domesticated phenotype (vigilance, fight off predators, search for food, periods of food shortage) to increased production (meat, milk, eggs, wool, reproduction). Reduced levels of activity, aggression, and a delayed and immature HPA axis response support that trend. Subsequently, production levels further increased with conscious selection for production traits. However, when resources become limited, it is expected that a further increase in production must result in further energy sparing on traits that are not directly selected for. Therefore, it can be hypothesized that under these conditions, selection for increased production may tend to, *vice versa*, further reduce aggression, activity, and the HPA axis response. In addition, selection for improved feed efficiency is expected to emphasize this trend because it specifically reduces the overall energy budget or metabolic scope.

Indeed, as reviewed by Rauw (2012), the literature shows a trend toward reduced activity with selection for improved feed efficiency, i.e., low residual feed intake or feed conversion ratio, or high feed conversion efficiency. Both Braastad and Katle (1989) and Luiting et al. (1991) showed that laying hens selected for low RFI were less active than those selected for high RFI. Efficient chickens were reported to spend more time resting than inefficient chickens (Morrison and Leeson, 1978; Katle et al., 1984). Meunier-Salaün et al. (2014) showed that a reduced physical activity in pigs from a line selected for low RFI contributed significantly to their improved feed efficiency. Sadler et al. (2014) observed that gilts from a line selected for low RFI spent less time standing, more time sitting, and were less active overall than pigs from a control line, and Colpoys et al. (2014) showed that low RFI male pigs were less active than those from a high RFI line. In growing cattle, variation in physical activity was estimated to account for approximately 10% of the variation in RFI (Herd et al., 2004).

In addition, the literature suggests a reduced fear response with selection for feed efficiency. Richardson and Herd (2004) report a positive genetic relationship between blood cortisol concentration of steers and their sire’s breeding value estimates for RFI, and a significantly lower blood cortisol concentration in steers selected for low RFI compared to steers selected for high RFI. Aleri et al. (2016) also showed that plasma cortisol concentrations in cattle were lower in high feed conversion efficiency cow phenotypes than in low feed conversion efficiency phenotypes 48 h post-yarding and handling. Chickens selected for low RFI in the study of Luiting et al. (1994) had a lower cortisol response to an ACTH challenge, albeit for a longer period, than chickens selected for high RFI. Efficient chickens have been reported to show a lower sensitivity to environmental disturbances than inefficient chickens (Morrison and Leeson, 1978; Katle et al., 1984). In pig, Colpoys et al. (2014) showed that male pigs from a line selected for low RFI displayed a shorter duration of freezing, froze less frequently, and attempted to escape less frequently than high-RFI pigs (Colpoys et al., 2014). In the same pig lines, Sadler et al. (2014) showed that gilts from the low-RFI line tended to have lower baseline cortisol concentrations and were less responsive to an ACTH challenge than gilts from the high-RFI line (Jenkins et al., 2013). In sheep, Knott et al. (2008) also observed that low-RFI sheep had a lower increase in cortisol concentration following an ACTH challenge.

Selection for juvenile body weight in poultry reduced rates of aggressive interactions in the study of Marsteller et al. (1980). Schütz and Jensen (2001) suggest that selection for feed efficiency in laying hens resulted in a concomitant reduction in social interactions saving energy that could be reallocated to production traits. Indeed, high efficient hens in the study of Braastad and Katle (1989) showed less escape and aggressive behavior than low efficient hens. However, according to Kjaer and Mench (2003) selection for increased egg production and concomitant acceleration of maturity and the onset of lay has also shown to result in animals that are socially more dominant and more aggressive than unselected hens. Likewise, although it is observed that livestock animals with a calm temperament have higher average daily gains than those with excitable temperaments (Voisinet et al., 1997; Holl et al., 2010), selection for high lean gain in pigs has also favored genetic lines with increased incidence of porcine stress syndrome (PSS) and individuals that have increased levels of fear and anxiety and more excitable temperaments. In these animals, the altered muscle properties responsible for faster lean growth also altered the response of muscle to the process of conversion to meat, leading to pale and soft exudative (PSE) and dark, firm, and dry (DFD) pork (Pajor et al., 2000; Lonergan et al., 2001; Adzitey and Nurul, 2011). In addition, social (aggressive) behavior of group-housed animals depends not only on the genotype of the individual but also on that of its group members (Wade et al., 2010). In this context, selection for individual performance of production efficiency may favor dominant (aggressive) individuals with higher resource acquisition and faster growth. Indeed, as reviewed by Muir (2003) selection based on individual bird productivity is associated with increased social dominance and aggressiveness when animals are housed in groups, resulting in higher rates of mortality and a negative response in group performance. Instead, group selection for production traits, taking into account competitive interactions of group members, reduced agonistic activity including feather pecking and cannibalism, improved feather scores, increased whole-blood serotonin concentrations, reduced fear-related behavior, and improved ability to withstand social, handling and environmental stress (Muir, 2003; Bolhuis et al., 2009; Ellen et al., 2014). In pigs, in one generation of selection for indirect genetic effects, selected pigs performed less non-reciprocal biting and showed considerably less aggression at reunion with familiar group members after they had been separated during a 24 h regrouping test (Camerlink et al., 2013).

The results suggest that selection for improved production efficiency, i.e., increased production on reduced feed intake, may be regarded as further selection for the domestic phenotype, thereby further reducing aggression, activity, and the fear response in domesticated livestock. However, the trend may depend on the genotype and the (social) environment in which the animals are selected. This hypothesis can be tested by following correlated traits in a selection experiment for production efficiency in livestock species, including measures of production efficiency, activity, aggressive behavior, fear response, HPA axis activity, and serotonin concentration. Several of the aforementioned studies suggest that selection for feed efficiency may improve stress-coping abilities and result in an animal welfare benefit in terms of calmer animals that are less reactive to novelty (e.g., Richardson and Herd, 2004; Colpoys et al., 2014; Aleri et al., 2016). However, because glucocorticoid hormones have been found to *strengthen* the adaption processes to stressors, including newborn survival, resistance to bacteria and parasites, and tolerance to heat stress (Mormède et al., 2010), further selecting for the domesticated phenotype may not necessarily be beneficial in all aspects.

## Synthesis and Conclusion

Coping styles, defined as a coherent set of behavioral and physiological stress responses that are consistent over time and characteristic to a certain group of individuals, have been described both in humans and in other animal species, and both in response to stressors in a natural environment, as well as in response to stressors in human-controlled production environments. Because coping styles function to reduce, minimize, master, or tolerate the internal and external demands of a stressful event, they are directly related to individual fitness, i.e., they are part of the life-history strategy.

A basic assumption of life-history strategy is the concept of energy budgets and energetic trade-offs between life-history traits. Behavioral styles trade off with other life-history traits through the acquisition and allocation of resources. Increased allocation costs of resources to employment of a coping style can result in greater fitness when this results in greater resource acquisition and/or reproductive success. However, in the production environment, domestication and subsequent artificial selection for production traits specifically focused on selection of individuals with energy sparing mechanisms for non-production traits. First, domestication resulted in animals with low levels of aggression and activity, and a low HPA axis reactivity. These animals were easier to handle and resources needed for these processes could now be invested into higher productive and reproductive outputs. We propose that, *vice versa*, selection for improved production efficiency may to some extent continue to favor docile domesticated phenotypes.

It is hypothesized that both domestication and selection for improved production efficiency result in the selection of predominantly reactive style animals. Both domesticated and reactive style animals are characterized by low levels of aggression and activity, and increased serotonin neurotransmitter levels. However, there is a discrepancy with respect to the HPA response, i.e., whereas domestication quite consistently results in a decrease in the functional state of the HPA axis, the reactive coping style is often found to be dominated by a *high* HPA response. The discrepancy may be resolved by distinguishing coping styles from personality variables. Our hypothesis may support the need to map the coping response along two independent underlying dimensions: coping behavior and fearfulness. Although the two dimensions often appear to be related, the latter in reality may mediate the coping response independently, as influenced by perception and appraisal of the stress event, which in turn is influenced by characteristics such as personality, emotion, motivation, and perception of control. In contrast to natural selection, domestication specifically involved animals that display a combination of low aggression *and* low fearfulness. As difficult as it is to quantify emotion and appraisal in animals, this suggests that coping response should not be equated to a concept of personality. Although it is generally proposed that animal welfare improves with selection for calmer animals that are less fearful and reactive to novelty, others have warned that animals bred to be less sensitive with fewer desires may be undesirable from an ethical point of view. In other words, extreme genetic modification of animals into “senseless, emotionless machines” that have no desires may be viewed as morally problematic (D’Eath et al., 2010; Rauw and Gomez Raya, 2015).

## Author Contributions

All authors conceived the article subject and developed the concepts during the draft. WR wrote the manuscript. AJ, LG-R, and JD commented on the drafts. All authors accepted the final version of the manuscript.

## Conflict of Interest Statement

The authors declare that the research was conducted in the absence of any commercial or financial relationships that could be construed as a potential conflict of interest.
